# A primary rat neuron-astrocyte-microglia tri-culture model for studying mechanisms of neurotoxicity

**DOI:** 10.3389/ftox.2024.1523387

**Published:** 2025-01-10

**Authors:** Jessie R. Badley, Aishwarya Bhusal, Pamela J. Lein

**Affiliations:** Department of Molecular Biosciences, School of Veterinary Medicine, University of California, Davis, CA, United States

**Keywords:** cortical neurons, *in vitro* model, neuroinflammation, neurotoxicity, rat

## Abstract

Primary cell cultures from rodent brain are widely used to investigate molecular and cellular mechanisms of neurotoxicity. To date, however, it has been challenging to reliably culture endogenous microglia in dissociated mixed cultures. This is a significant limitation of most *in vitro* neural cell models given the growing awareness of the importance of interactions between neurons, astrocytes and microglia in defining responses to neurotoxic exposures. We recently developed a tri-culture model consisting of neurons, astrocytes and microglia dissociated from the developing rat neocortex and demonstrated that this tri-culture model more faithfully mimics *in vivo* neuroinflammatory responses then standard neuron-only or neuron-astrocyte co-cultures. Here, we describe our protocol for generating tri-cultures of rat cortical neurons, astrocytes and microglia in which all 3 cell types can be maintained for up to 1 month in culture at the same relative ratio observed in the developing rat neocortex. We also discuss applications of this model for neurotoxicity testing, as well as the potential of this model to fill a current gap for assessing neuroinflammation in the *in vitro* testing battery for developmental neurotoxicity.

## 1 Introduction

Neurotoxicity is an important regulatory endpoint for protecting human health; however, studies conducted following current regulatory guidelines (e.g., USEPA, OECD), which largely rely on *in vivo* rodent models, are not available for the majority of chemicals to which humans are exposed (Krewski et al., 2020). This reflects the fact that *in vivo* models of neurotoxicity are expensive, labor- and time-intensive, and require large numbers of animals (Lein et al., 2005; Bal-Price et al., 2012). To address the paucity of neurotoxicity data, there is currently significant research investment in developing and validating new approach methodologies, including *in vitro* methods, for assessing the neurotoxic hazards of chemicals and for elucidating the mechanism(s) by which neurotoxic chemicals interfere with function and/or development of the nervous system (Avila et al., 2020; Tal et al., 2024).

Crosstalk between neurons, astrocytes, and microglia plays a crucial role in maintaining neural homeostasis, and one of its key functions is to trigger neuroinflammatory responses. While neuroinflammation normally functions to protect the brain from diverse insults, chronic neuroinflammation can be detrimental to brain health, and is implicated in the etiopathogenesis of numerous neurodevelopmental and neurodegenerative diseases (Ibrahim et al., 2020; Kim et al., 2020; Park et al., 2024). Aberrant neuroinflammation can disrupt neuronal circuit formation during critical developmental periods, which is associated with autism spectrum disorder and schizophrenia (Fan et al., 2023), while persistent activation of microglia and/or astrocytes can promote neuronal damage and death, which is thought to contribute to disease progression in Alzheimer’s and Parkinson’s disease (Gao et al., 2023).

Neuroinflammation is increasingly implicated as a neurotoxic mechanism by which diverse chemotypes contribute to adverse neurological outcomes across the human lifespan (Harry and Kraft, 2008; Kraft and Harry, 2011). Environmental toxins, such as traffic-related air pollution (Levesque et al., 2011; Patten et al., 2021), organophosphate pesticides (Andrew and Lein, 2021) and metals (Blaylock, 2012; Bondy, 2021), have been shown to activate neuroinflammatory pathways, and these neuroinflammatory responses coincide with chemical-induced phenotypes with face validity to neurodevelopmental disorders and neurodegenerative diseases.

There is significant interest in developing *in vitro* models for screening chemicals to assess effects on neuroinflammation and for studying neuroinflammatory mechanisms of neurotoxicity. However, it has been challenging to model neuroinflammation *in vitro* because most of the standard models either lack microglia or consist of purified microglial populations. Thus, many widely used neural cell culture models fail to fully capture the complex interactions between microglia, neurons, and astrocytes. A robust *in vitro* model is needed to better understand which chemicals promote or inhibit neuroinflammatory responses, and the mechanisms by which they do so.

**FIGURE 1:**
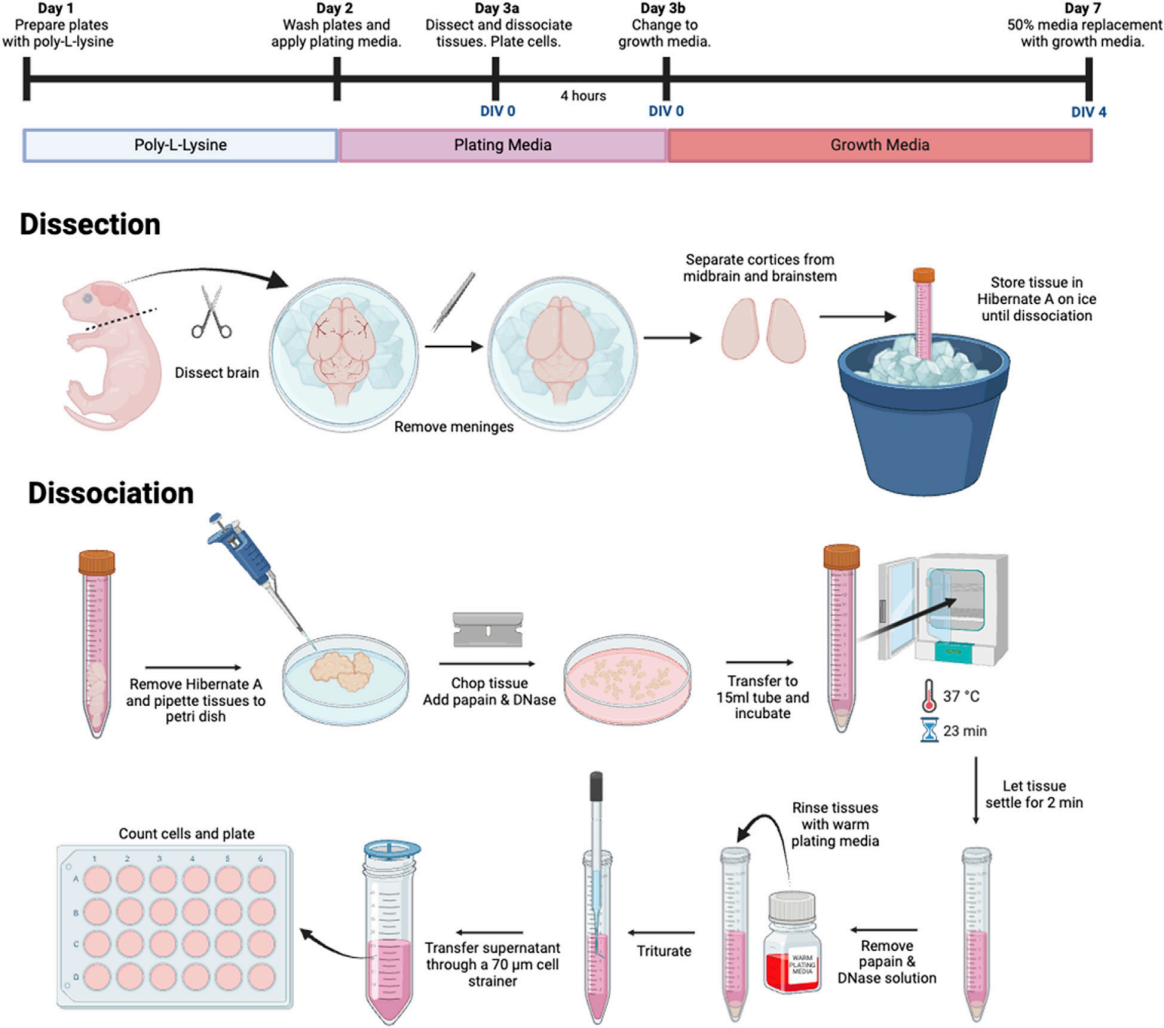
Flowchart summary of primary tri-culture dissection and dissociation procedure.

**FIGURE 2:**
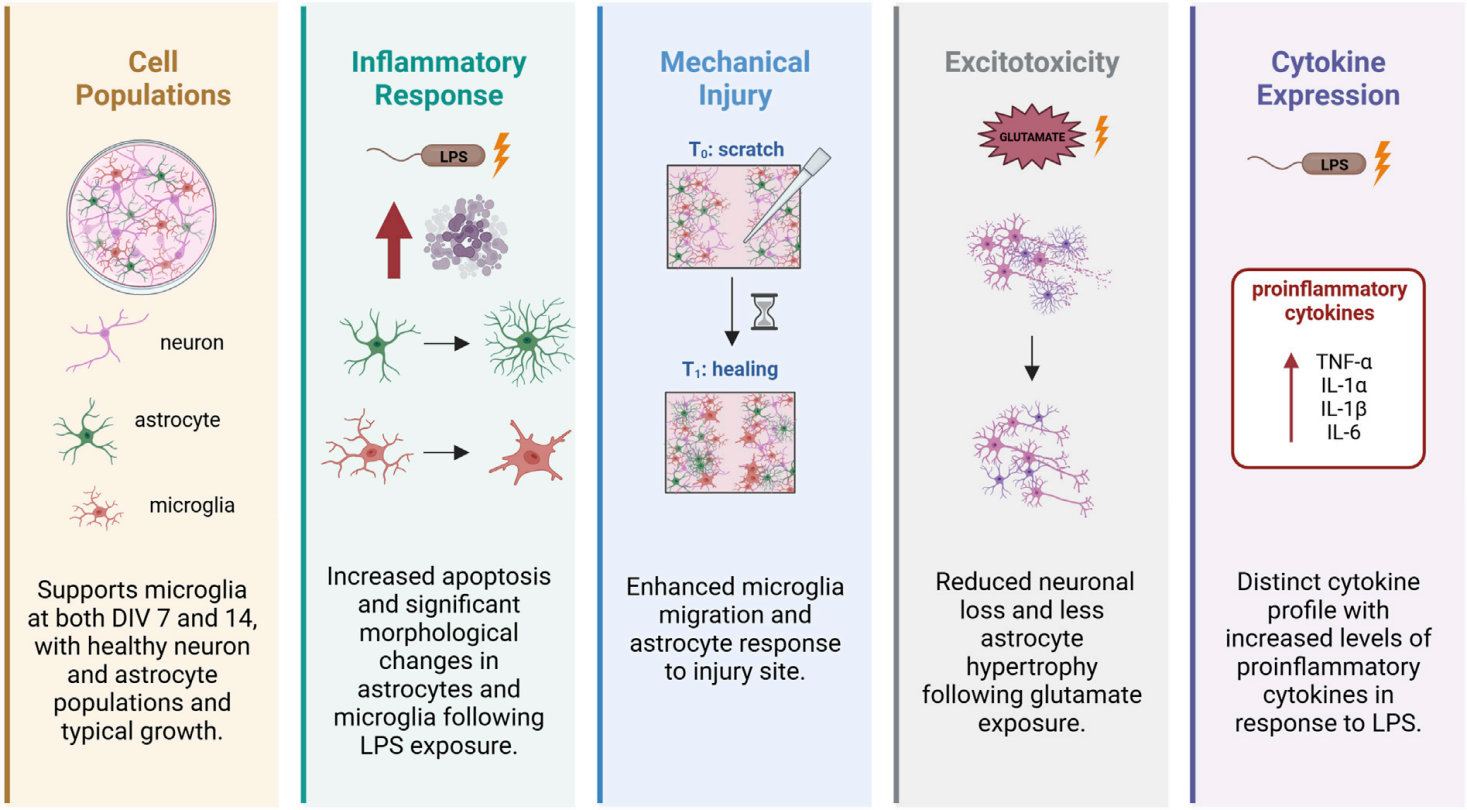
Microglia modulate astrocyte and neuronal responses as evidenced by enhanced inflammatory and neuroprotection in tri-culture model vs. neuron-astrocyte co-culture model. Adapted from Goshi et al. (2020).

Here, we provide a detailed description of our recently established protocol for a primary tri-culture model consisting of neurons, astrocytes and microglia dissociated from the developing rat neocortex (Figure 1). This tri-culture model has been previously shown (Goshi et al., 2020) to replicate *in vivo* neuroinflammatory responses more accurately than standard neuron-only or neuron-astrocyte co-cultures (Figure 2). For example, in response to the proinflammatory stimulus lipopolysaccharide (LPS), the tri-culture model exhibited increased caspase 3/7 activity, astrocyte hypertrophy, and secretion of pro-inflammatory cytokines, such as TNF-α, IL-1α, IL-1β, and IL-6 compared to neuron only and neuron-astrocyte co-cultures (Figure 2). Additionally, the inclusion of microglia in the tri-culture model provided neuroprotection against glutamate-induced excitotoxicity, which was not observed in the neuron only vs. neuron-astrocyte co-cultures, highlighting the importance of microglia in mediating neuroinflammatory responses and maintaining neuronal health. These results suggest that the tri-culture model may serve as a valuable *in vitro* tool for studying neuroinflammation as a neurotoxic response, and that this model may fill the current gap for assessing neuroinflammation in the *in vitro* testing battery for developmental neurotoxicity (Blum et al., 2023).

## 2 Materials and equipment

### 2.1 Materials

• 12 mm #1 German glass coverslips (Electron Microscopy Services, 72196-12).
• 24 well plates (flat bottom, Fisherbrand, FB012929).
• 70 μm cell strainer (Fisherbrand, 22363548).
• 96 well plates (black, plastic; Costar, 3603).
• Bent-tip 16-gauge needle (BD PrecisionGlide, 305176).
• Brain dissection instruments.
  ⁃ large decapitation scissors (Fine Science Tools, 14501-14).
  ⁃ micro scoopula/spatula (Millipore, Z648299).
  ⁃ spring scissors (Fine Science Tools, 15006-09).
  ⁃ medium tweezers (Fine Science Tools, 11000-16).
  ⁃ microdissection tweezers (Dumont by Fine Science Tools, #4, #5).
• Cellometer Auto T4 counter and chambers (Nexcelom Bioscience, SD100) or hemocytometer.
• CO_2_ Incubator (ThermoFisher Scientific, 3130).
• Container for pups (a freezer box lined with paper towels is fine).
• Dissection light (Chui Technical Lumina).
• Dissection microscope (Nikon SMZ-10A).
• Disposable pipet (Fisher, 12-711-9AM).
• Fire-polished bent-tip pipettes (fire-polished in-house).
• Fluid aspiration vacuum system (BVC Profressional) (VacuuBrand, 20727503).
• Germinator 500 Glass Bead Sterilizer (Cellpoint, 5-1450).
• Ice bucket (Corning, CLS432123).
• Inverted Phase Contrast Microscope (Nikon, TS100).
• Laminar Flow Hood (Baker, EG6320).
• Microscope slides (frosted, Fisher, 1255015).
• Paper towels (doused with 70% EtOH).
• Plastic 150 mm × 15 mm TC-treated Petri dishes (Fisher, FB0875714).
• Plastic 60 mm × 15 mm TC-treated Petri dishes (Corning, 430166).
• ProLong Gold Mountant with DAPI (Invitrogen, P36931).
• Several 15 mL conical tubes (Falcon, 352096).
• Several 50 mL conical tubes (Fisher Scientific, 14-955-239).
• Spray bottle.
• Steri-flip filter tube (Millipore, SCGP00525).
• Sterile steel back razor blades (Personna, 94-120-71).
• Tube racks.
• Whatman filter paper (Cytiva, 1002-090).
• Wide-tip tweezers.

### 2.2 Reagents

• B-27 plus supplement (×50) (Gibco, A3582801).
• Borax (Sigma B3545).
• Boric acid (Sigma B7660).
• Bovine serum albumin (Sigma-Aldrich, 0000303356).
• Dulbecco’s Phosphate-Buffered Saline, calcium, magnesium (DPBS++) (×10) (Gibco, 14080-055).
• Ethyl Alcohol (EtOH, Koptec, V1001).
• Ethyl Alcohol, Absolute, Reagent: 200 Proof (Spectrum, 6RFK9).
• GlutaMAX Supplement (×100) (Invitrogen, 35050061).
• Hanks Balanced Salt Solution (×10) (Gibco, 14065-056).
• Heat-inactivated horse serum (Invitrogen, 26050-088).
• HEPES buffer agent (Fisher, BP310-500).
• Hibernate A (Gibco, A1247501).
• MilliQ water (Millipore system).
• Neurobasal Plus Media (Gibco, A3582901).
• Ovine wool cholesterol (Avanti Polar Lipids, 700000P).
• Papain, lyophilized (Worthington BioChem, LS003119).
• Paraformaldehyde (Sigma, 441244).
• Poly-L-Lysine hydrobromide (Sigma, P1399).
• Sodium hydroxide (NaOH, Sigma, S-0899).
• Sodium phosphate buffer (Na_2_HPO_4_, Sigma, S9763; NaH_2_PO_4_, Sigma, S9638).
• Recombinant Human TGF-β1 (HEK293 derived) (PeproTech, 100-21).
• Recombinant Mouse IL-34 Protein (R&D Systems, 5195-ML-010; or BioLegend, 577604)
• Triton X-100 (Fisher, 9002-93-1).

### 2.3 Preparation of cell culture media, substrates, and other solutions

#### 2.3.1 Plating medium

Components:

• 500 mL Neurobasal Plus Media.
• 10 mL B27 Plus supplement (×50).
• 5 mL GlutaMAX Supplement (×100).
• 50 mL Heat-inactivated horse serum.
• 10 mL HEPES buffer (1M, pH 7.5).

Instructions:

• Sterile filter and store at 4°C for up to 2 months.

#### 2.3.2 Triculture growth medium

Components:

• 500 mL Neurobasal Plus Media.
• 10 mL B27 Plus supplement (×50).
• 5 mL GlutaMAX Supplement (×100).
• 100 ng/mL Mouse IL-34.
• 2 ng/mL TGF-β1.
• 1.5 μg/mL Ovine Wool Cholesterol.

Instructions:

• Sterile filter and store at 4°C. Make fresh weekly.

#### 2.3.3 Mouse IL-34

Components:

• 10 µg of IL-34
• 2 mL of 1x DPBS++ containing 0.1% (w/v) Bovine Serum Albumin.

Instructions:

• Reconstitute IL-34 in 1x DPBS++ to achieve a final stock concentration of 5,000 ng/mL.
• Prepare 1 mL aliquots. Each aliquot is used to prepare 50 mL of Triculture Growth Medium.

#### 2.3.4 TGF-β1

Components:

• 2 µg of TGF-β1
• 20 mL of 1x DPBS++ containing 0.1% (w/v) Bovine Serum Albumin.

Instructions:

• Reconstitute TGF- β1 in 1x DPBS++ to achieve a final stock concentration of 100 ng/mL.
• Prepare 1 mL aliquots.
• Each aliquot is used to prepare 50 mL of Triculture Growth Medium.

#### 2.3.5 Ovine wool cholesterol

Components:

• 2.5 mg of Ovine Wool Cholesterol.
• 0.5 mL of Absolute ethanol.
• 66.17 mL of MilliQ water.

Instructions:

• Dissolve the Ovine Wool Cholesterol in Absolute ethanol.
• Add dissolved cholesterol to MilliQ water to achieve a final concentration of 75 μg/mL.
• Prepare 1 mL aliquots.
• Each aliquot is used to prepare 50 mL of Triculture Growth Medium.

#### 2.3.6 Borate buffer

Components:

• 3.1 g Boric Acid.
• 4.75 g Borax.
• ∼1 L MilliQ water.

Instructions:

• Prepare the buffer in a tissue culture safe container to ensure there is no residue.
• Add Boric Acid and Borax to 750 mL MilliQ water and mix until dissolved.
• Once dissolved, add MilliQ water to 1 L.
• Adjust the pH to 8.5.
• Sterile filter and store at room temperature.

#### 2.3.7 Dissection media

Components:

• 500 mL of MilliQ water.
• 25 mL of HEPES buffer (1M, pH 7.55).
• 50 mL Hanks Balanced Salt Solution (×10).

Instructions:

• Sterile filter the media.
• Store at 4°C for up to 2 months.
• If not used frequently, prepare multiple 50 mL aliquots and store half at −20°C and half at 4°C.

#### 2.3.8 Hibernate A

Instructions:

• Mark the bottle with initials and date of opening, and designate it as “sterile”.
• Store at 4°C.
• Prepare 10 mL aliquots on the day of dissection according to the protocol.

#### 2.3.9 Poly-L-Lysine (PLL), 0.5 mg/mL

Components:

• 200 mg of Poly-L-Lysine (PLL).
• 40 mL of Borate buffer.
• 360 mL of MilliQ water.

Instructions:

• Weigh out PLL into a 50 mL conical tube and note the exact amount.
• Add 40 mL of Borate buffer and shake at room temperature to dissolve PLL.
• Sterile filter dissolved PLL and add enough of MilliQ water to achieve a final concentration of 0.5 mg/mL PLL (360 mL for 200 mg of PLL).
• Store at 4°C for up to 2 months.

#### 2.3.10 DNase, 2 mg/mL

Components:

• 100 mg DNase, lyophilized.
• 50 mL DPBS++ (×1).

Instructions:

• Dissolve 100 mg of DNase into 50 mL of DPBS++ (×1) to achieve a final concentration of 2 mg/mL DNase.
• Sterile filter and prepare 500 mL aliquots.
• Store in −30°C.
• Each aliquot is used to prepare 10 mL of Papain/DNase solution.

#### 2.3.11 Sodium phosphate buffer, 0.2M

Components:

• 35.6 g Na_2_HPO_4_.
• 27.6 g NaH_2_PO_4_.
• MilliQ water, 1 L each.

Instructions:

• Dissolve the Na_2_HPO_4_ and NaH_2_PO_4_ separately in ∼750 mL of MilliQ water.
• Once dissolved, adjust the volume of each stock solution to 1 L.
• Store each stock solution separately at room temperature for up to 2 months.
• To prepare 25 mL of buffer, mix together 18 mL of Na_2_HPO_4_ stock solution and 7 mL of NaH_2_PO_4_ stock solution.

#### 2.3.12 Paraformaldehyde, 4% (w/v)

Components:

• 8 g Paraformaldehyde (PFA).
• 10 mM NaOH solution (500 µL of 2 M NaOH into 100 mL of MilliQ water).
• 25 mL of 0.2 M Sodium Phosphate Buffer.

Instructions:

• Prepare a 10 mM NaOH solution.
• Inside of a fume hood, dissolve 8 g of PFA into the 10 mM NaOH solution while heating to 55°C and stirring. *Note: any hotter will create a toxic PFA vapor.*
• Once dissolved and at 55°C, filter the solution through Whatman filter paper and prepare 25 mL aliquots stored in 50 mL conical tubes.
• Store at −20°C until needed or at 4°C for up to 1 week.
• Mix 25 mL of 8% PFA with 25 mL of 0.2 M Sodium Phosphate Buffer.
• If using a frozen 8% PFA aliquot, ensure it is fully thawed, dissolved, and back in solution before adding the 0.2 M Sodium Phosphate buffer. Thaw aliquot at 4°C overnight and then transfer to a 37°C water bath to dissolve any remaining crystals.

#### 2.3.13 Fire-polished bent-tip glass pipette

Instructions:

• Use a standard 9-inch glass pipette and carefully fire-polish the tips under a dissection microscope.
• The edge should be smooth, with the tip diameter no smaller than ∼85% of its original size.
• Wash after each use, stuff with cotton, and autoclave pipettes after use.
• If cellular debris accumulates inside the pipette, it can be cleaning with a 0.5 M hydrochloric acid bath before additional use.

## 3 Methods

### 3.1 Rat pups

Timed-pregnant rat dams (*Rattus norvegicus*) are purchased from Charles River Laboratory (Hollister, CA). Primary cortical neuron-astrocyte-microglia tri-cultures can be isolated from postnatal day 0 (P0) to P2 rat pups.

### 3.2 Day 1: Plate preparation

1. Place Sterile Coverslips.
  • Transfer coverslips into each well of a 24-well plate.
  • Use a sterile plastic pipette tip (without filter) with a vacuum to drop each coverslip into a well.
  • Turn down the vacuum valve to minimal flow to facilitate easy transfer.
2. Add 0.5 mg/mL PLL.
  • Total Volume per well:
    ⁃ 24-well plates: 250 µL.
    ⁃ 96-well plates: 50 µL.
  • Ensure the PLL covers all surfaces of the coverslips.
3. Incubate Plates with PLL.
  • Incubate with PLL overnight in a 37°C Tissue Culture incubator.
  • Note: This step is usually done overnight, however, you may wash the plates and add Plating Medium on the same day as long as the PLL has coated the plates for 2 h.

### 3.3 Day 2: Wash plates

1. Aspirate out the PLL.
  • Tilt plates away from you.
  • Aspirate all of the PLL from each well with a vacuum.
2. Wash Plates Three Times.
  • Use room temperature sterile MilliQ water for washing.
  • Completely coat the coverslips with sterile MilliQ water and shake the plates to ensure thorough washing.
  • Aspirate all of MilliQ water out with a vacuum.
3. Apply Plating Medium.
  • Incubate with Plating Medium overnight in a 37°C Tissue Culture incubator.
  • Total Volume per well:
    ⁃ 24-well plates: 500 µL.
    ⁃ 96-well plates: 100 µL.
  • Place plates in a 37°C Tissue Culture incubator for at least 3–4 h, preferably overnight, to allow Plating Medium components to bind to the plate.

### 3.4 Day 3: Day of dissection–day *in vitro* 0

#### 3.4.1 Initial set up

1. Prepare workspace with the following:
  • Sterilize dissection tools and instruments with glass bead dry sterilizer.
  • Ice bucket.
  • Waste/Biohazard bag for carcasses.
  • Paper towels sprayed with 70% EtOH.
  • Four 60 mm × 15 mm plastic Petri dishes.
  • One 150 mm × 15 mm plastic Petri dish.
2. Prepare a 50 mL conical tube with Papain.
  • Weigh out 23 mg of lyophilized Papain to a 50 mL conical tube.
  • Add 10 mL of Hibernate A and warm in a 37°C water bath inside an incubator during the dissection.
3. Thaw DNase.
  • Remove DNase aliquot from −30°C to thaw at room temperature.
4. Warm Medias.
  • Put Plating Medium and Triculture Growth Medium into a 37°C incubator to warm during the dissection.
5. Set up Dissection Microscope and Light.
  • Place a plastic 150 mm × 15 mm TC-treated Petri dish containing crushed ice on the stage.
6. Prepare Dissection Media and Hibernate A.
  • Place dissection media into 60 mm × 15 mm plastic Petri dishes and place on top Petri dish with ice on dissection microscope stage.
  • Preparate 10 mL aliquots of Hibernate A in conical tubes (e.g., one for each sex and/or microdissected brain region) and place in an ice bucket.

#### 3.4.2 Dissection of neocortices: 1–3 h total working time, depending on skill level

Note: Pups can be sex-segregated at this point, if needed.

1. Decapitate pup(s).
  • On top of the 70% EtOH-soaked paper towels, use the large decapitation scissors to cut between the chin and the shoulders.
  • Line up the head(s) and spray well with 70% EtOH. Spray your gloves with 70% EtOH, as well.
2. Expose the brain.
  • Grab the head with the medium tweezers at the sides of the jaw. Using spring scissors, gently cut through the skin and skull from the caudal end to between the eyes.
  • Note: Pull up the spring scissors while cutting to preserve brain structure.
3. Remove the brain.
  • Use a scalpel/scoopula to gently sever the optic nerve with a back-and-forth motion, and then lift the brain and transfer it into the Petri dish containing dissection media over ice on the stage.
4. Repeat the above steps for the remaining pups.
5. Separate the Neocortices from the Midbrain/Brainstem.
  • Orient the brain dorsally.
  • Use curve-tipped forceps to hold the brain at the cerebellum and the #5 forceps to separate the cortices along the longitudinal fissure.
  • Pry one cortex up to expose the midbrain/brainstem connecting tissue and blunt dissect the neocortex. Repeat on the other side.
6. Remove the meninges and blood spots.
  • Use one set of tweezers to hold the optic nerve and the other to peel back the meninges. Remove as many blood spots as possible to avoid cell culture debris.
7. Transfer cortices to conical tubes.
  • Once all the brains have been dissected, transfer the cortices from dissection media-containing Petri dish (es) to their respective conical tubes containing Hibernate A on ice.
8. Complete the dissection.
  • Repeat the above steps until all the brains have been dissected.

#### 3.4.3 Dissociation of neocortices: 1–2 h total working time

1. Prepare Papain/DNase solution.
  • Add 500 µL aliquot of thawed DNase to the warmed, dissolved Papain and Hibernate A solution.
  • Sterile filter using a Steri-flip.
2. Transfer the tissue(s) to a new 60 mm × 15 mm plastic Petri dish.
  • Transfer the micro dissected brain regions to a new Petri dish, being careful to minimize Hibernate A transfer. Let the tissue settle in the Petri dish, and aspirate any excess Hibernate A if necessary.
3. Mince tissue(s).
  • Using a sterile razor blade, mince the tissue with quick strokes. Spin the dish while mincing to ensure evenness.
  • Repeat for all tissues, using a new sterile razor blade for each.
4. Transfer tissue(s) to conical tube.
  • Add the Papain/DNase solution to the minced tissue(s) in the Petri dish (es) and transfer to new 50 mL conical tube(s).
  • Split the solution between each tissue, using *at least* 2 mL per tissue. I.e.: 10 mL between sexes, 5 mL each.
5. Incubate with Papain/DNase solution.
  • Incubate at 37°C for 23 min, mixing the tissues every 3–5 min by lightly shaking the conical tube.
  • Note: Do NOT leave the tissue in the Papain/DNase solution for any longer than 23 min.
6. Neutralize the Papain/DNase solution.
  • Neutralize the Papain/DNase solution with equal parts Plating Medium.
7. Let the tissue settle.
  • Let the tissue settle for 2 min. Aspirate the neutralized solution, being careful not to disturb cells at the bottom of the conical tube.
8. Rinse and triturate the tissue.
  • Add 10 mL of warm Plating Medium to the tissue in the 50 mL conical tube. Prime the fire-polished pipette with Plating Medium, and then triturate 20–30 times until the suspension becomes cloudy.
9. Filter the cell suspension.
  • Let the tissue particles settle, and then transfer the upper cloudy suspension to a new 50 mL conical tube by filtering through a 70 μm cell strainer. Leave the last 1 mL to avoid transferring large tissue particles.
10. Repeat steps 8 and 9.
  • Repeat trituration once more, until no large tissue chunks remain.
  • Note: Be careful not to over-triturate, there should be some tissue/cellular debris that remains at the bottom of the conical tube when done with this step.
11. Combine and count the cells.
  • Mix the combined supernatants by gently pipetting up and down, then count the cells using a hemocytometer or automated cell counter.

#### 3.4.4 Plating the cell suspension: ∼5 h total working time, including a 4 h incubation period

1. Determine the volume of cell suspension needed per well.
  • Using the cell count, determine the volume of cell suspension needed per well and transfer cells to plates. Dilute the cell suspension if needed.
  • If preparing many plates, use an aliquotter to speed up the process.
2. Distribute and allow cells to attach.
  • Shake the plates lightly to evenly distribute the cells over the coverslips.
  • Return the plates to the CO_2_ tissue culture incubator for 3–4 h to allow cells to attach to the coverslips.
3. Change the media.
  • After the cells have attached, gently tap and shake the plates to dislodge any debris and aspirate out the Plating Medium. Replace with Triculture Growth Medium, applying slowly to avoid dislodging cells.
    ⁃24-well plates: 500 µL.
    ⁃96-well plates: 100 µL.

#### 3.4.5 Cell culture feeding and maintenance: every 3–4 days

1. Remove one-half volume from each well.
  ⁃24-well plates: 250 µL.
  ⁃96-well plates: 50 µL.
2. Add one-half volume of fresh, warmed Triculture Growth Medium to bring the volume back up.
  • Repeat every 3–4 days until plates are used for experiments.

#### 3.4.6 Statistical analysis

• For all experiments, a minimum of three biological replicates or independent dissections should be used, with a minimum of three technical replicates per each biological replicate or independent dissections.

## 4 Results

Cholesterol, IL-34, and TGF-β, the three supplements in the tri-culture media, have been shown to support isolated microglia and are found in astrocyte-conditioned media (Bohlen et al., 2017). An exploratory study of whether neurons or astrocytes constitutively secrete these compounds into the tri-culture was negative; thus, addition of all three supplements is required for a healthy tri-culture of neurons, astrocytes, and microglia (Goshi et al., 2020).

### 4.1 Neurons, astrocytes, and microglia are supported in long-term tri-cultures

**FIGURE 3:**
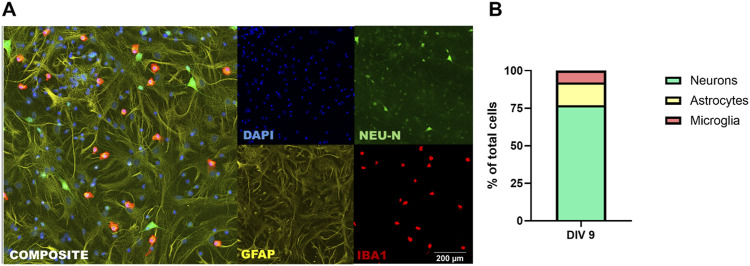
Tri-culture medium supports neurons, astrocytes, and microglia. **(A)** Representative fluorescent photomicrographs of a DIV 9 tri-culture immunostained for neurons (NeuN; green), astrocytes (GFAP; yellow) and microglia (Iba1; red). All cultures were counterstained with DAPI (blue) to visualize nuclei. Scale bar = 200 µm. **(B)** Percentage of neurons, astrocytes and microglia of total cell population at DIV 9 as determined using automated high content imaging (ImageXpress) and image analysis (MetaXpress).

To quantify neurons, astrocytes and microglia in the tri-culture, cultures can be fixed with 4% (w/v) PFA solution in PBS and then immunostained for cell type-specific antigens, e.g., β-III tubulin or NeuN to identify neurons; GFAP, astrocytes; and Iba1, microglia. Consistent with previously reported results (Goshi et al., 2020), quantitative analysis of NeuN, GFAP and Iba1 immunoreactive cells as the percentage of the total number of cells in the culture, as determined by the number of DAPI-labeled nuclei, indicated that there are significant populations of neurons, astrocytes, and microglia at DIV 9 (Figure 3A). In tri-culture conditions (Figure 3B), the neurons constituted ∼80% of the total cell population; astrocytes, ∼10–12%; and microglia, ∼7–8%. These proportions closely resemble the cell type distributions observed *in vivo* (Savchenko et al., 2000; von Bartheld et al., 2016).

Primary cell cultures derived from the neocortex at P0 predominantly consist of excitatory pyramidal neurons but can also include a small proportion of other neuronal subtypes, such as interneurons. These cultures are incredibly versatile tools for modeling a wide range of neurodevelopmental and neurodegenerative diseases because of their ability to recapitulate many fundamental properties and processes like dendritic and axonal growth, neurodegeneration, excitability, and responses to chemical and physical insults. These tricultures have been used by several laboratories to model Alzheimer’s disease (Luchena et al., 2022; Kim et al., 2024) and Parkinson’s disease (Zhang et al., 2024).

While small fluctuations in cell percentages are inevitable due to biological variability inherent in individual animal brains, our data suggest that the overall proportions remain reproducible within 10%–20% under the described experimental conditions. Furthermore, the inclusion of three technical replicates per condition per experiment and careful statistical analysis (e.g., two-way ANOVA) ensure that variability is rigorously quantified and accounted for.

### 4.2 Endpoints that can be measured in the tri-cultures

#### 4.2.1 Neuroinflammation

To mimic bacterial infection, cultures can be challenged with LPS (3.0 × 10^6^ EU/mg; Sigma) reconstituted in DPBS++ (final concentration 5 μg/mL). When exposed to LPS on DIV 7 following a media change, tri-cultures exhibit several characteristics of neuroinflammation, including elevated caspase 3/7 levels, morphological changes consistent with activation in both astrocytes and microglia, and release of pro-inflammatory cytokines (Goshi et al., 2020). Although neurodegeneration and neurite loss are also observed in the LPS-exposed tricultures, the damage is significantly less severe compared to *in vivo* observations (Goshi et al., 2020).

To simulate neuroinflammation triggered by mechanical injury, a scratch test can be performed by dragging a sterile 200 µL pipette tip across the bottom of each well in cultures on DIV 7. This fairly common technique is used to not only simulate mechanical injury, but also to measure cell migration. Microglia migrate into the scratch in the tri-culture model, comparable to what is observed *in vivo*; however in the tri-culture, the migrating microglia do not necessarily have larger surface area than microglia in the non-scratched control condition (Goshi et al., 2020).

Microglia can also play a significant neuroprotective role during excitotoxic events. When challenged with glutamate, tri-cultures have significantly less neuronal cell loss compared to neuron-astrocyte co-cultures. To trigger excitotoxicity using glutamate, a fresh 50 mM solution of L-glutamic acid (Sigma) in DPBS++ can be used. On DIV 7, remove and save half of the medium from each well and store at 37°C. Dilute the glutamate solution to varying concentrations (1:100) directly into each well; vehicle controls should receive an equal volume of sterile DPBS++. Incubate the glutamate-exposed cultures at 37°C for 1 h. At the completion of the incubation, remove the medium from each well and replace it with the respective conditioned media and proceed to add 250 µL of fresh media for a “media change”. Unlike exposure to LPS or simulated mechanical injury, exposure to glutamate does not induce a change in microglia morphology (Goshi et al., 2020).

#### 4.2.2 Apoptosis

In response to LPS, the tri-culture model displays many classic hallmarks of neuroinflammation, including an increase in caspase 3/7 activity. Apoptosis can be quantified using the Caspase-Glo^®^ 3/7 Assay System (Promega) according to the manufacturer’s protocol by measuring luminescence using a H1 hybrid microplate reader (BioTek Instruments).

#### 4.2.3 Calcium imaging

The tri-culture model has been confirmed to be electrophysiologically active as determined via calcium imaging (Goshi et al., 2020). Cultures were loaded with the cell-permeant calcium indicator, Fluo-4 a.m. (ThermoFisher) following the manufacturer’s protocol. Calcium fluxes were measured in response to the addition of DPBS++ with or without varying concentrations of L-glutamic acid (Sigma) added. Prior to the addition of glutamate, a ×200 magnification fluorescence image was obtained of each well. Following a 2-min incubation with the glutamate solution, a second fluorescence image was captured of the same field-of-view and same exposure time. The change in fluorescence intensity before vs. after glutamate was calculated.

#### 4.2.4 Cytokine profile

A significant number of pro-inflammatory cytokines are found to be significantly increased in the conditioned media of tri-cultures after LPS challenge, including TNF-α, IL-1α, IL-1β, and IL-6. These same cytokines are consistently secreted by microglia in response to LPS in a variety of experimental conditions and are often used as biomarkers to indicate neuroinflammatory or neurodegenerative disorders. Following the DIV 7 media change, cultures were exposed to 5 μg/mL LPS or vehicle for 48 h. Following incubation, the conditioned media was collected, centrifuged to remove any cells and the supernatant stored at − 80°C until analysis.

### 4.3 Advantages and disadvantages

One of the major advantages of the tri-culture model is that it preserves the relative ratio of neurons, astrocytes and microglia observed *in vivo*. Thus, the tri-culture model provides an *in vitro* platform for studying the influence of crosstalk between neurons, astrocytes and microglia on neurotoxic responses. It also provides a model for screening chemicals for effects on neuroinflammation, both chemical-induced neuroinflammation and chemical inhibition of neuroinflammatory responses (e.g., to LPS or mechanical injury).

However, it is important to note that the tri-culture model does not capture interactions with blood-brain barrier components, oligodendrocytes or systemic immune cells, which are increasingly implicated in the modulation of neuroinflammation and response to neurotoxic agents (Bernardino et al., 2024). Additionally, previous studies of the cytokine profile of the tri-culture model suggests that even under control conditions, the cultures are in a mildly inflamed state (Goshi et al., 2020). Furthermore, the use of IL-34 to support microglial survival may limit accessibility of this model since it is a relatively costly reagent that is not always readily available.

### 4.4 Potential pitfalls and troubleshooting

Several potential pitfalls that impact the quality and/or reproducibility of this model include low cell yield, reduced cell viability and culture contamination. Low cell yield and reduced viability typically result from issues with dissection and/or cell dissociation steps. The primary problem with the dissection is taking too long to dissect the appropriate brain region following euthanasia. Keeping the euthanized pups in ice-cold media, dissecting brain tissues on ice, and practicing the dissection so it can be rapidly performed will help mitigate this issue. To mitigate compromising the yield and/or viability during the dissociation step, tissues must be handled gently, and freshly prepared enzyme solutions should be used.

## 5 Discussion

There is increasing evidence that neurotoxic agents trigger neuroinflammatory responses in both the developing and adult brain, but to date it has been challenging to study these effects *in vitro* because most cell culture models do not support the three neural cell types critically involved in forming and regulating the neuroinflammatory response, e.g., neurons, astrocytes and microglia. The tri-culture model described here represents a physiologically relevant platform for studying the interactions between neurons, astrocytes and microglia in response to neurotoxic stimuli. For example, his tri-culture system has been used by other laboratories to study nitric oxide-induced necroptosis (Zhang et al., 2024) and the effects of ion channel blockers on neuronal excitability (Phadke et al., 2022). We are currently using the tri-culture model to study the cellular and molecular mechanisms of PCB developmental neurotoxicity. Our preliminary observations indicate that the dendritic response of cortical neurons to PCB 95 varies in the co-culture vs the tri-culture model. Ongoing studies are determining the concentration-effect relationships in both culture models and in neuron-only culture models to determine whether inclusion of microglia is required to recapitulate the dendritic retraction observed *in vivo* in weanling rats exposed developmentally to PCBs in the maternal diet and then subjected to training in the Morris water maze (Yang et al., 2014).

## Data Availability

The raw data supporting the conclusions of this article will be made available by the authors, without undue reservation.
